# The jewel wasp *Nasonia vitripennis* utilizes two single-copy, protamine-like sperm nuclear basic proteins

**DOI:** 10.1093/g3journal/jkag066

**Published:** 2026-03-20

**Authors:** Patrick C Zhang, Kassandra Soriano Martinez, Gaby Negrete, Findley Finseth, Juan Ausio, Patrick M Ferree

**Affiliations:** Department of Natural Sciences, Pitzer College and Scripps College, Claremont, CA 91711, United States; Department of Natural Sciences, Pitzer College and Scripps College, Claremont, CA 91711, United States; Department of Natural Sciences, Pitzer College and Scripps College, Claremont, CA 91711, United States; Department of Natural Sciences, Pitzer College and Scripps College, Claremont, CA 91711, United States; Department of Biochemistry and Microbiology, University of Victoria, Victoria, BC V8W 2Y2, Canada; Department of Natural Sciences, Pitzer College and Scripps College, Claremont, CA 91711, United States

**Keywords:** sperm nuclear basic proteins, protamine-like proteins, chromatin, reproductive development, spermatogenesis, sexual conflict, jewel wasp, hymenopteran insects

## Abstract

The DNA of sperm is uniquely packaged into an exceptionally condensed chromatin state with sperm nuclear basic proteins (SNBPs). These proteins are diverse, including protamines, protamine-like (PL) proteins, testis-specific (TS) histones, and high mobility group (HMG)-box proteins. Usage of SNBP type varies widely among and within phyla. Moreover, SNBPs can be evolutionarily dynamic in copy number and amino acid composition, even among closely related species. Despite the diversity of insects, little is known about SNBP usage across members of this taxonomic group. A previous study biochemically identified SNBP candidates from the male reproductive tissue of the jewel wasp, *Nasonia vitripennis*, in addition to other insects. Here, we computationally examined existing *N. vitripennis* tissue transcriptomes to independently identify SNBP candidates. Our analyses uncovered 13 genes encoding proteins with SNBP characteristics, including exclusive gene expression in the testis, short protein length, and a high proportion of basic residues. The 2 highest-expressed of these genes encode PL proteins, which are evolutionary derivatives of the histone H1 family. Targeted degradation of the transcripts produced by the highest-expressed PL gene resulted in partial male sterility and defective sperm nuclear morphology in fertilized embryos, while RNAi treatment of the other PL gene yielded a subtle fertility effect. Both genes are present across hymenopteran insects but not outside this insect order. Moreover, they are not evolutionarily dynamic like the testis-specific HMG-box SNBPs in *Drosophila* species, which may reflect the lack of sex chromosomes, and the resulting conflict between them, in hymenopteran insects.

## Introduction

The chromatin of sperm is exceptional because it is packaged into a uniquely condensed state for organization within the particularly small nuclear volume of this cell type. This packaging enables the sperm's hydrodynamic function; protects its genome from oxidative and other types of damage, especially in insects and other organisms in which sperm are retained for long periods in the female's reproductive tract after copulation; and prevents premature division of the sperm's nuclear material upon entry into the egg's cytoplasm (Braun 2001; Balhorn 2007; Nili et al. 2009; Fortes et al. 2014; Dubruille et al. 2023, 2025). Establishment of sperm chromatin occurs during the final phase of spermatogenesis, when most of the conventional histones are removed from DNA and replaced with highly basic, sperm nuclear basic proteins (SNBPs) (Rousseaux et al. 2008; Rathke et al. 2014). Following fertilization, SNBPs are rapidly removed by factors in the egg cytoplasm, and the sperm's DNA is immediately repackaged with histones for compatibility with the egg's nuclear material (Loppin et al. 2015).

Remarkably, formation of sperm chromatin is achieved through the use of different SNBP types across the higher eukaryotes (Bloch 1976; Ausió 1999). Some vertebrates including primates and certain fish such as salmon express very small genes encoding protamines (Prots), short (50 to 110 aa length) proteins that contain as much as 80% arginine residues (Bloch 1976; Lewis et al. 2004a). The highly positive charge of Prots allows them to strongly interact with DNA, countering its electronegative charge, thereby facilitating tight chromatin compaction (Balhorn 2007). The Prots of some eutherian mammals also contain cysteines that enable inter- and intraprotamine disulfide bond formation, further aiding chromatin compaction (Balhorn 1982). Many other organisms, especially invertebrates, instead express protamine-like (PL) proteins. These proteins are derived evolutionarily from histone H5, a replication-independent member of the histone H1 family that is expressed in terminally differentiated cells (Lewis 2003; Eirín-López et al. 2006a). PLs are similar in structure to proteins in this family. For example, they contain a globular head region that was originally defined as being resistant to trypsin digestion (Hartman et al. 1977; Ausio et al. 1987). A defining feature of this globular region, in both PLs and histone H1, is a winged-helix (WH) domain that facilitates association with chromatin by interacting with the major groove of linker DNA between the nucleosomes (Saperas et al. 2006). Additionally, like histone H1, the C-terminal half of a PL protein is highly disordered. However, the PL contains a stretch of 30 to 50 amino acids in this region that is rich in arginine and lysine residues and resembles a Prot. In some organisms that utilize PLs, including some mollusks and tunicates and a liverwort, the short arginine-/lysine-rich region is posttranslationally cleaved from the rest of the protein (Carlos et al. 1993; Lewis et al. 2004b; D’Ippolito et al. 2019), producing a single product, or multiple products in the case of the liverwort, which are thought to finalize condensation (Kasinsky et al. 2021). Here, we refer to such an excised, basic, and functional sequence as a PL peptide. It has been proposed that the process of PL association with DNA and its cleavage to produce DNA-interacting PL peptides underlies the liquid-liquid phase separation (LLPS)-like changes of the chromatin state that occur during the histone-to-SNBP transition (Kasinsky et al. 2021). While most examined organisms utilize either Prots or PLs to establish sperm chromatin, a handful of different organisms employ other proteins. Some frogs package sperm DNA with testis-specific histones (TS histones) (Alder and Gorovsky 1975), which presumably interact with sperm DNA similarly to their conventional histone counterparts. Alternatively, the fruit fly *Drosophila melanogaster* employs less basic SNBPs belonging to the B-subtype of the high mobility group (HMG) protein family for sperm DNA packaging (Chang et al. 2023). These SNBPs, including Mst35A and Mst35B, contain an HMG-box domain that may facilitate interactions with DNA or proteins (Malarkey and Churchill 2012). In the fruit fly, Mst35A and Mst35B may function together with a histone H1 family protein, Mst77F, to package sperm DNA in this insect (Jayaramaiah Raja and Renkawitz-Pohl 2005).

Why is there such a range of different SNBPs that facilitate sperm chromatin formation? This question is particularly compelling considering 2 additional patterns. First, certain major taxonomic groups are not uniform in their usage of SNBP type. For example, while salmon employ Prots, other fish including pike, mackerel, and sharks use PLs (Bloch 1976), and still other members of this group use TS histones (Saperas et al. 1994). Similar heterogeneous usage of SNBPs also has been observed within other natural groups (Bloch 1976), suggesting that SNBP genes, at least in certain cases, are labile over evolutionary time. Second, regardless of the type, SNBPs tend to be among the most rapidly evolving genes in an organism. This pattern is true for the 2 mammalian Prot genes *prm1* and *prm2* (Torgerson et al. 2002; Lüke et al. 2014, 2016a, 2016b), as well as the sperm-specific HMG-box SNBPs in *Drosophila* species (Chang et al. 2023). Of particular interest is understanding the forces that drive SNBP turnover and sequence divergence. In the case of PRM2, certain changes in amino acid composition, including an increase in arginine residues, were found to be positively correlated in certain lineages with longer sperm head length, which may positively affect sperm function (Lüke et al. 2014). In fruit fly species, gene turnover and rapid evolution of HMG-box SNBPs, especially those that are sex linked, may be driven by genetic conflict between the sex chromosomes (Chang et al. 2023 ). Testing the universality of these explanations will require broader analyses across a wider range of organisms with and without sex chromosomes.

Aside from SNBP studies in *D. melanogaster* (Jayaramaiah Raja and Renkawitz-Pohl 2005; Alvi et al. 2013; Tirmarche et al. 2014; Kimura and Loppin 2016; Chang et al. 2023), little is known about these proteins in insects, despite the great evolutionary breadth of this major animal order. A previous study characterized 2 germline-specific histone H1 variants, PpH1V1 and PpH1V2, which appear in the nuclei of elongating sperm in the parasitoid wasp, *Pteromalus puparum* (Yuan et al. 2023). Targeting PpH1V1 with RNA interference resulted in male sterility, making it an SNBP candidate. A more recent study employed a biochemical approach to identify candidate SNBPs in several insects including the honeybee, *Apis mellifera*, and another parasitoid wasp, *Nasonia vitripennis* (jewel wasp) (Leyden et al. 2024). For *A. mellifera*, 2 highly basic peptides were purified from mature sperm. One of these peptides, named Am-P1, is very arginine rich and appears to be processed from a PL protein that contains a WH domain near the N-terminus. For *N. vitripennis*, 5 peptides were obtained from a limited amount of adult male reproductive tracts. Three of these peptides perfectly matched genes in the *N. vitripennis* genome. The other 2 wasp peptides were unidentifiable. Because the sequences of the jewel wasp and honeybee peptides differed substantially, it was concluded that these insects utilize different SNBPs. However, no determination was made in this study on the identities of the wasp genes and the characteristics of their encoded proteins. While biochemical purification has been a highly successful approach for identifying SNBPs, this strategy may not recover low-abundance proteins, especially when tissue amounts are limited, or when the use of pure sperm is not feasible for protein discovery.

As an independent and complementary approach, we have computationally examined existing, tissue-specific *N. vitripennis* transcriptomes to identify candidate SNBP genes in this insect. *N. vitripennis* has become an important experimental model organism for several areas in hymenopteran biology, including reproductive development, comparative genomics, and host–symbiont interactions (Werren and Loehlin 2009). Identifying the complete set of *N. vitripennis* SNBPs would facilitate functional studies of these proteins with modern tools available in this insect, including systemic RNA interference (sRNAi) (Lynch and Desplan 2006) and CRISPR-mediated genome editing (Li et al. 2017; Zhang et al. 2024). Our work produced a set of 13 wasp SNBP candidates. Two of these genes match peptides identified in the biochemical study, making them of highest interest. The bioinformatical characteristics of the 2 *N. vitripennis* genes suggest that both encode PLs, which we have named Nv-PL1 and Nv-PL2. Nv-PL1 is orthologous with the *A. mellifera* PL protein, Am-PL1, and the *P. puparum* SNBP candidate, PpH1V1. Nv-PL2 appears to have a different origin. By comparison across wasp species, both *N. vitripennis* PLs are much more divergent within their C-terminal disordered regions than in their WH domains, consistent with rapid divergence of SNBPs observed in other organisms. Targeting each PL with sRNAi resulted in measurable male sterility and dysfunctional sperm chromatin observed in newly fertilized embryos. Nv-PL1 is present in all hymenopteran insects examined, while Nv-PL2 is present in most, and neither was found outside Hymenoptera, suggesting that these genes are unique to this insect order. Finally, these PL genes are not evolutionarily dynamic in duplication and loss like the male-specific HMG-box genes in *Drosophila* species.

## Methods

### Bioinformatical identification and characterization of *N. vitripennis* SNBPs

Previously published *N. vitripennis* transcriptomes were used to identify SNBP candidates (Akbari et al. 2013; Ferree et al. 2015). These transcriptomes were generated from 4 different adult tissue conditions: (i) whole male reproductive tracts, (ii) male carcasses (without reproductive tracts), (iii) whole ovaries, and (iv) female carcasses (without ovaries). These datasets were cross-compared to find protein-coding genes that were exclusively expressed in the male reproductive tract. We deemed that, for a given gene to be exclusively expressed in the male reproductive tract, it must have 100 or more reads per kilobase per million (RPKM) in the male reproductive tract and no more than 5 RPKM in any of the other tissue conditions. From the resulting list of testis-exclusive, protein-coding genes, isoelectric point values were calculated using the Isoelectric Point Calculator (IPC) service (Kozlowski 2016) and assigned to each. To assign gene ontology (GO) or functional terms, we used previously annotated names. For uncharacterized or unassigned genes, we used the Blast2Go pipeline (Gotz et al. 2008), with the initial BLAST conducted using DIAMONDS (Buchfink et al. 2021) and the following parameters (–ultra-sensitive –outfmt 6 – max-hsps 1 –evalue 1e-10). Further protein assignments were conducted using interPro's InterProScan with default settings (Philip et al. 2014). Scripts for determining protein sequence length, amino acid composition, and arginine placement were written in Python (Supplementary Table 2). 3D protein predictions were generated using AlphaFold V2.0 DB (Jumper et al. 2021).

### Estimating sequence evolution rates

DIAMONDS was used to identify orthologs in nucleotide format for the orthologs of the Nv-PLs. Nucleotide sequences were converted into amino acids using the EMBOSS transeq tool (Rice et al. 2000), and Seqtk (GitHub—lh3/seqtk: Toolkit for processing sequences in FASTA/Q formats 2025) was used for sequence formatting. Gene sequences were aligned using MAFFT (Katoh and Standley 2013). Pal2Nal (Suyama et al. 2006) was used to generate and format files for phylogenetic analyses by maximum likelihood (Yang 1997). PAML control files were generated following a previously published protocol (Álvarez-Carretero et al. 2023). The one-ratio codeml site model (M0) was used for the purpose of calculating an evolutionary rate for the WH region of Nv-PL1 and Nv-PL2. Due to alignment difficulties, values could not be generated for the PL peptide portions of the genes.

### Genetic testing of SNBP candidates using systemic RNA interference

sRNAi was conducted following a previous procedure (Ferree et al. 2026). As an overview of this procedure, a 500- to 800-bp region of the coding sequence of Nv-PL1 and Nv-PL2 was amplified from complementary DNA (cDNA) made from total testis RNA. Forward and reverse primers used for PCR contained the T7 viral promoter sequence. PCR products were purified using the Qiaquick PCR purification kit (Qiagen catalog number 28104) and subsequently used as a template for bidirectional in vitro transcription with the MEGAscript RNAi kit (Thermo Fisher Scientific catalog number AM1626). After clean-up, double-stranded RNA (dsRNA) was microinjected into wild-type male pupae in the yellow body developmental stage. Once the dsRNA-treated males reached adulthood, they were crossed with wild-type females in a pairwise manner (1 male with 1 female). Each male–female pair was placed into a separate vial and set on 2 fresh *Sarcophaga bullata* host pupae. Females were allowed to oviposit into the hosts for 2 full days before removal. Hosts containing wasp progeny were placed into a 25 °C incubator until emergence of F1 progeny, at which point brood sex ratios were scored.

### Wasp embryo collection, fixation, and microscopic analysis

Wild-type females were crossed with sRNAi-treated males *en masse* (∼30 females and the same number of males) and allowed to oviposit into fresh *S. bullata* hosts for 2 h at 25 °C. The young embryos were removed from the hosts and placed into a glass vial with lid, and fixative ingredients were rapidly added in the following order and amounts: 3-mL heptane, 1.5-mL 1× PBS (phosphate-buffered saline), and 600-µL 37% paraformaldehyde. The embryos were fixed on a platform rocker for exactly 30 min. Subsequently, embryos were removed from the fixative, dried briefly, and devitellinized while immobilized on a double-sided sticky tape in 1× PBT (PBS with 0.1% Triton-X 100 detergent) using a small (30 gauge) needle.

Fixed embryos were mounted in Vectashield medium (Vector labs catalog number H-1200-10) containing 4′,6-diamidino-2-phenylindole (DAPI). Imaging was conducted on a Leica SPE DMIRB inverted fluorescence confocal microscope. Collected Z series images were collapsed and exported as JPEG files. Figures containing microscopic images were built using Adobe Photoshop.

## Results

### Computationally identifying SNBPs in *Nasonia*

As an independent means of identifying *N. vitripennis* SNBPs, we analyzed existing wasp transcriptomes (Akbari et al. 2013; Ferree et al. 2015). These datasets were specific for (i) the whole male reproductive tract, (ii) the ovary, (iii) the male carcass (without the male reproductive tract), and (iv) the female carcass (without the ovary). Given the specialized roles of SNBPs in packaging sperm DNA (Balhorn 2007), we reasoned that SNBP genes should be expressed only in the male reproductive tract. By comparing these datasets, we identified 417 TS protein-coding genes (out of 15,266 total genes in the Nvit_2.1 wasp genome) (Supplementary Table 1). Because this set of genes likely includes all male-specific genes needed to produce functional sperm, we filtered them using SNBP-related criteria. For example, because SNBPs range from moderately to strongly basic, we removed from this list all genes whose encoded proteins have predicted isoelectric points below 8.0. This threshold allowed us to identify any TS histones, which tend to be less basic than Prots or PLs. This filtering step left 231 genes, to which we assigned gene ontology (GO) terms. We removed any protein-coding genes with GO terms that are not reflective of SNBPs, such as “electron transport chain,” “mitochondria,” “microtubule-associated,” and “kinase,” leaving only those with terms such as “DNA-associated,” “histone or histone-like,” “chromatin-associated,” “nucleosome-associated,” and “uncharacterized.” This last level of filtering yielded a final set of 13 genes (Table 1).

**Table 1.** A summary of 13 SNBP candidates after bioinformatic filtering.

Gene ID	GO names	Expression (RPKM)	Isoelectric point
LOC100116183 (Nv-PL1)	**C**: nucleosome **F**: DNA binding **P**: nucleosome assembly	232,063	10.7
LOC100118976 (Nv-PL2)	**C**: nucleosome **F**: DNA binding **P**: nucleosome assembly	44,374	11.5
LOC100120046	**C**: nucleus, chromosome **F**: DNA binding, protein tyrosine phosphatase activity, protein tyrosine/serine/threonine phosphatase activity, methyltransferase activity, zinc ion binding **P**: chromatin organization, protein dephosphorylation, regulation of exit from, anatomical structure morphogenesis, histone lysine methylation, response to chemical, neuron development, cell division	18,950	9.5
LOC100678761	**C**: nucleus **F**: DNA binding, NAD+ ADP-ribosyltransferase activity, zinc ion binding, transferase activity, glycosyltransferase activity, metal ion binding **P**: obsolete protein ADP-ribosylation, nitrogen compound metabolic process, macromolecule metabolic process, primary metabolic process	14,643	8.4
LOC100114216	**C**: nucleus **F**: DNA-binding transcription factor activity, zinc ion binding, sequence-specific DNA binding **P**: regulation of DNA-templated transcription	12,063	8.1
LOC103316131	Uncharacterized	5,086	10.7
LOC103316943	**C**: nucleus **F**: DNA binding	4,967	10.7
LOC116417036	**C**: membrane **F**: nucleic acid binding **P**: DNA integration	2,598	8.1
LOC116416958	**P**: DNA biosynthetic process	1,197	8.2
LOC103316653	**C**: membrane, sarcoplasmic reticulum **F**: calcium channel activity **P**: regulation of DNA-templated transcription, histone modification, calcium ion transmembrane transport	1,002	9.3
LOC100119399	**C**: nucleosome, extracellular region, nucleoplasm **F**: DNA binding, structural constituent of chromatin, protein heterodimerization activity **P**: nucleosome assembly, telomere organization, epigenetic regulation of gene expression	635	11.5
LOC103316712	**P**: DNA biosynthetic process	598	8.8
LOC107980474	**C**: transcription factor TFIIH core complex, chromosome **P**: nucleotide-excision repair, chromatin remodeling, transcription initiation at RNA polymerase II promoter	149	9.2

LOC numbers are given for each gene. Expression levels in the male reproductive tract are shown in RPKM (reads per kilobase of transcript per million mapped reads).

### *Nasonia* expresses SNBP candidates with PL characteristics

**Fig. 1.**
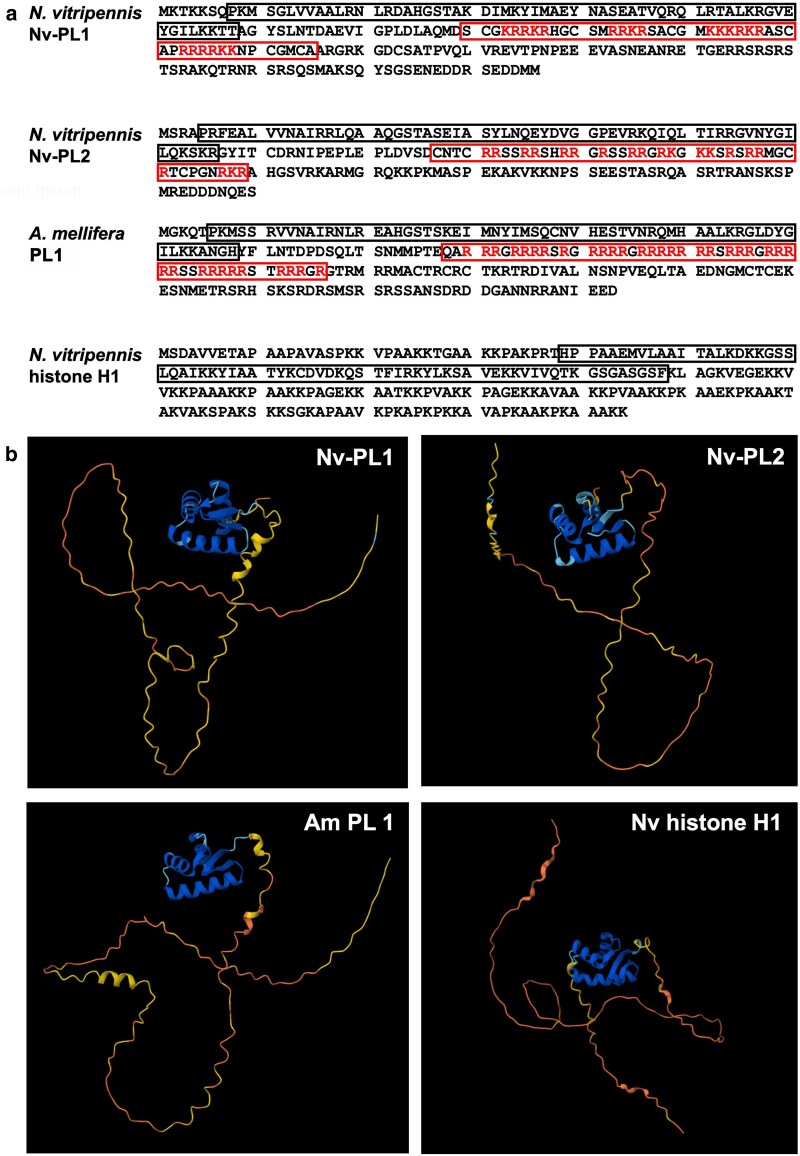
The top 2 *N. vitripennis* SNBP candidates are similar in sequence and predicted 3D structure to the PL protein, Am-PL1, of *A. mellifera*, and histone H1. a) Primary amino acid sequences are shown for Nv-PL1, Nv-PL2, Am-PL1, and *N. vitripennis* histone H1. The first box in each protein represents the WH domain. The second box in each PL denotes the previously identified peptide in the disordered region. b) AlphaFold 3D structures of Nv-PL1 and Nv-PL2, as well as of Am-PL1 and histone H1 of *N. vitripennis*. The short alpha helices of the WH domain in each structure are blue.

We bioinformatically scrutinized these 13 genes to assess which, if any, are likely to encode SNBPs. None of these genes had characteristics of Prots (i.e. exceptionally short open reading frames/proteins with a very high arginine content). To avoid missing any Prots, we examined the unfiltered TS set of genes for those with these characteristics, again finding no such Prot candidates. However, the 2 highest-expressed genes in our final list of 13 genes encode proteins that perfectly matched 2 of the *N. vitripennis* peptides that were previously purified biochemically (Leyden et al. 2024). The higher-expressed gene of these 2, LOC100116183, encodes a 216 aa protein containing a region near its N-terminus that resembles a WH domain, reminiscent of the one present in the globular head of histone H1. The C-terminal half of the protein is predicted to be unstructured (Fig. 1). Within this unstructured region resides one of the previously identified peptides, which contains multiple arginine and lysine residues as well as several cysteines. The next highest-expressed gene, LOC100118976, encodes a protein with a very similar amino acid length, sequence composition, and predicted structure to that of LOC100116183 (Table 1; Fig. 1), even though these 2 proteins do not appear to share homology, as assessed by a lack of clear sequence alignment. Interestingly, the predicted proteins of these 2 genes strongly resemble the *A. mellifera* PL in overall structure (Fig. 1). These characteristics suggest that these 2 *N. vitripennis* genes are PLs, and like Am-PL1, the wasp PLs are likely to be posttranslationally cleaved to produce the previously identified, highly basic peptides that are present in mature sperm. Given these findings, we henceforth refer to LOC100116183 as Nv-PL1 and LOC100118976 as Nv-PL2.

Of the 11 remaining genes in our filtered list, 2 are also of potential interest. Like the other 2 PL candidate genes, LOC103316131 has an N-terminal WH domain and an unstructured C-terminal half containing a highly basic region (Supplementary Fig. 1). Another gene, LOC103316943, contains an HMG-box domain at its C-terminus. Interestingly, this latter protein has a similar pI value and tertiary structure to the HMG-box proteins encoded by the *D. melanogaster* genes, Mst35A (ProtA) and Mst35B (ProtB), which are not as basic as PLs. A third gene, LOC100119399, encodes a H3.1-like protein; conventional histone H3.1 functions in constitutive heterochromatin in other organisms (Hake and Allis 2006; Jacob et al. 2014). We were unable to identify a true H3.1 in the *N. vitripennis* genome, but the H3.1-like gene is most similar to the replication-independent protein H3.3A (92% similarity; BLAST E-value of 6 × 10^−96^). Due to the low expression level of this gene in *N. vitripennis* (Table 1; Supplementary Table 1), it was deemed to be a poor SNBP candidate. The remaining 8 genes have predicted properties, indicating that they are not SNBPs (Table 1). Thus, they could play auxiliary roles in the histone-to-protamine transition, or they may instead function in other nonchromatin roles in the testis.

### Nv-PL1 and Nv-PL2 are single-copy genes that encode evolutionarily dynamic proteins

We sought to determine the distribution of Nv-PL1 and Nv-PL2 across organisms. Using BLAST, we searched for homologs of these genes in all archived genomes. Several patterns emerged from this work. First, for each gene, we found numerous matches across hymenopteran insects. Specifically, Nv-PL1 was found in 39 members, while Nv-PL2 was present in 33 of the same members (Supplementary Table 1). However, there were no matches outside Hymenoptera, arguing that these SNBPs, while ancient, are unique to this insect group. Second, BLASTing each *N. vitripennis* PL yielded different matching genes, and neither gene retrieved the other wasp SNBP despite their similarity in predicted 3D structure. The best matches for Nv-PL1 included uncharacterized genes and histone H1-like genes in both wasps and other nonwasp hymenopterans (Supplementary Table 1). In *A. mellifera*, Nv-PL1 best matched the Am-PL1 gene, and it was most similar to PpH1V1 in *P. puparum*. In contrast, Nv-PL2 was found to best match Luc7-like genes, histone H5-like genes, and those labeled as uncharacterized, in other hymenopteran insects (Supplementary Table 1). Third, each wasp PL retrieved a single matching gene for each hymenopteran insect, suggesting that the wasp genes have not undergone expansion within each species as has been the case for some of the HMG-box SNBPs in *Drosophila* species.

**Fig. 2.**
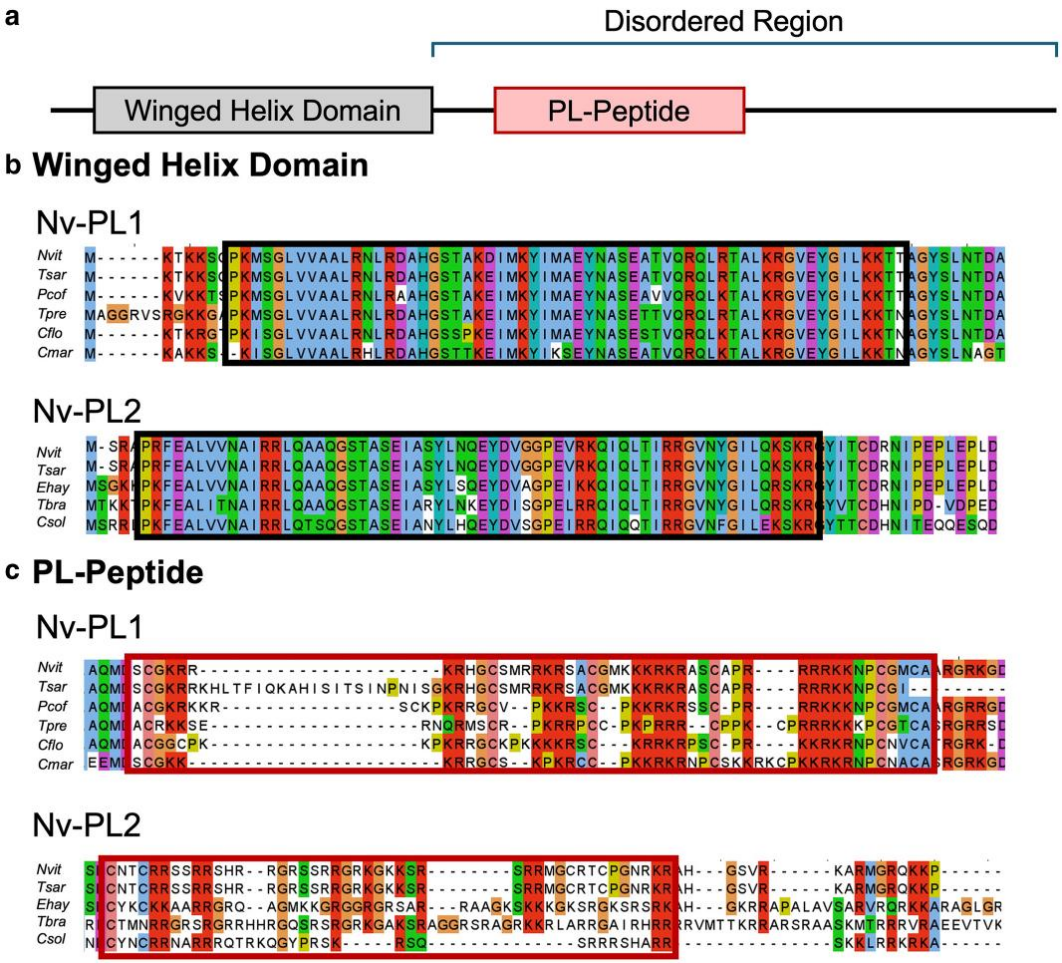
For both wasp SNBPs, the WH domain is highly conserved, while the highly basic PL peptide within the disordered region has low conservation. Comparisons are made in this figure among presumed orthologs from other wasps. a) A simple map depicting the general positions of the WH domain and the PL peptide region in the PL proteins. b) Alignment of the WH domain of Nv-PL1 and Nv-PL2 wasp orthologs (black boxed region), showing few polymorphisms in this region. c) Alignment of the PL peptide (red boxed region) within the disordered region of Nv-PL1 and Nv-PL2 wasp orthologs, showing a higher level of polymorphisms compared to the WH domain. The full genus and species names of the wasps for the orthologs shown are *Ceratosolen solmsi marchali (Cmar), Copidosoma floridanum (Cflo), Eretmocerus hayati (Ehay), Nasonia vitripennis (Nvit), Phymastichus coffea (Pcof), Trichogramma brassicae (Tbra), Trichogramma pretiosum (Tpre),* and *Trichomalopsis sarcophage (Tsar)*.

We next addressed if Nv-PL1 and Nv-PL2 are dynamic at the amino acid level, like the SNBPs of some other organisms (Torgerson et al. 2002; Lüke et al. 2014; Chang et al. 2023). Each of these proteins was aligned to their counterparts from other wasp species (Fig. 2) and, separately, to homologs from wasp and nonwasp hymenopterans (Supplementary Fig. 2). From these alignments, it became clear that the WH domains of these 2 genes are mostly unchanged among their orthologs (Fig. 2). A different pattern emerged regarding the unstructured, C-terminal halves of both proteins. In each case, this region was nearly identical between *N. vitripennis* and *Trichomalopsis sarcophagae*, its nearest known relative outside the *Nasonia* genus. However, between *N. vitripennis* and more distantly related wasps, there were numerous amino acid changes, as well as single and multiple amino acid insertions and deletions, in both genes (Fig. 2).

To measure the rate of evolution, we calculated omega (ω) values, which reflect nonsynonymous vs synonymous changes, for the WH domains of Nv-PL1 and Nv-PL2. This goal was possible because of the unambiguous alignment of this region across species. The ω value for this region of Nv-PL1 was 0.15, which is below the median omega value for all *N. vitripennis* genes (0.176) and substantially lower than the median value for TS genes (0.309). In contrast, the ω value of the same region of Nv-PL2 was 0.46, suggesting that Nv-PL2 has a more accelerated rate of evolution but is only slightly higher than the TS median. Although ω values of <1 technically indicate purifying selection, averaging ω values across genes can mask neutral or even adaptive evolution acting on a small number of sites due to purifying selection at most codons. We note that an average ω value of 0.46 is relatively high when calculated across a gene region. This elevated value may reflect positive selection acting on specific sites within the region. However, the limited number of available orthologs precludes robust site-based tests for positive selection. Calculating ω for the unstructured region was not possible for either PL given the uncertainty in sequence alignment due to the high number of amino acid gains and losses that are intermingled around and within the poly-arginine tracks. Regardless, this level of change, even without an ω value, demonstrates that the unstructured, C-terminal halves of both proteins, including the highly basic PL peptides, are highly dynamic, even within the wasp group (Fig. 2). Thus, both genes appear to be changing rapidly in this part of their encoded proteins.

### Targeting Nv-PL1 and Nv-PL2 with sRNAi results in male sterility and paternal nuclear defects following fertilization

**Fig. 3.**
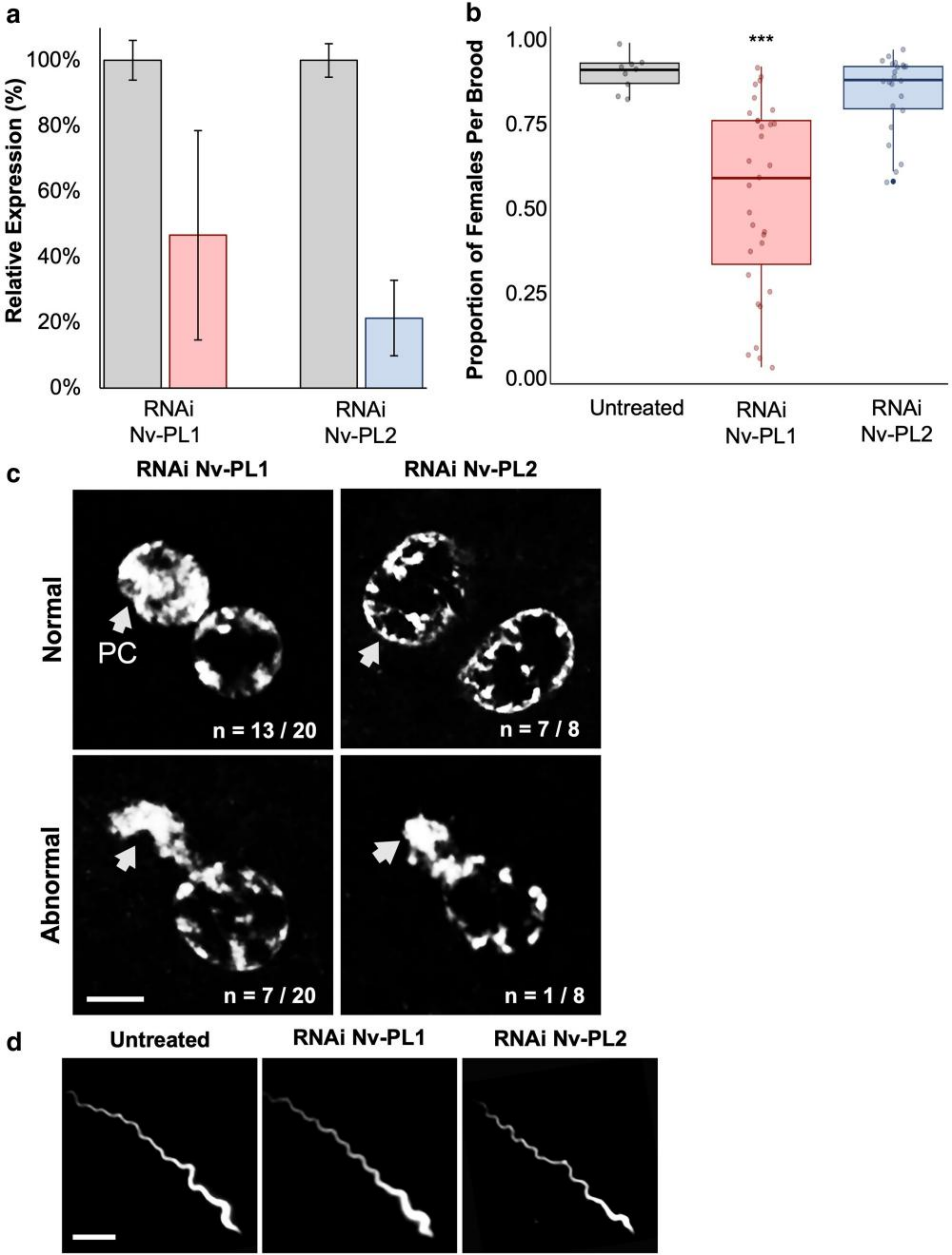
Systemic RNAi targeting of Nv-PL1 results in alteration of progeny sex ratio and a defective paternal nucleus in young embryos. a) Relative expression levels of sRNAi-targeted Nv-PL1 and Nv-PL2 transcripts as measured by RT-qPCR. b) Box plot diagrams showing distributions of sex ratios resulting from sRNAi-treated fathers. Lines represent median values, and boxes depict 1 SD. The asterisks denote statistical difference to 3 orders of magnitude, compared with the control (untreated) group. c) Confocal microscopic images of the paternal and material nuclei in 0- to 1-h embryos produced by sRNAi-treated fathers. In each image, the paternal nucleus is indicated by the white arrow. The scale bar is 4 μM. d) The nuclei of mature sperm from control and sRNAi-treated males. The scale bar is 3 μM. In (c) and (d), DNA is depicted in gray.

To functionally test Nv-PL1 and Nv-PL2, we used sRNAi to target the transcripts of these genes in males. Based on the likely identity of these genes as SNBPs, we predicted that sRNAi treatment would result in defective sperm nuclear function. To test this prediction, we first crossed sRNAi-treated males with untreated females and scored the sex ratios of their F1 progeny. Under normal circumstances, wild-type parents produce broods of progeny that are 80% to 90% females, reflecting the high fertilization rate of this insect and the fact that females arise as diploids from fertilized eggs, which inherit 2 nuclear sets. The degradation efficiency of Nv-PL1 and Nv-PL2 transcripts, measured by RT-qPCR, was 43% and 78%, respectively (Fig. 3a). We found that sRNAi-Nv-PL1-treated males sired broods of progeny ranging from 7% to 92% females, with a median of 54% (Fig. 3b). In contrast, the sex ratio for progeny produced by sRNAi-Nv-PL2-treated males was 58% to 95%, with a median of 84% (Fig. 3b). There was an overall significant difference in the proportion of females per brood when these distributions were compared to untreated broods (1-way ANOVA, F(2,60) = 20.2, *P* = 0.0001). A Tukey Honestly Significant Difference (HSD) test revealed that sRNAi-treated Nv-PL1 males sired significantly fewer F1 females per brood than Nv-PL2 males or untreated males (Tukey HSD *P* < 0.0001). This effect would be expected to result from fertilized embryos that end up developing as haploids due to loss of a defective paternal set. The proportion of females per brood produced by Nv-PL2 and untreated males did not differ significantly from those sired by untreated males (Tukey HSD *P* = 0.505), although there was a slight downward trend in the sex ratio.

To investigate the nature of the sRNAi effect on Nv-PL1, we microscopically inspected 0 to 1-h embryos sired by sRNAi-Nv-PL1-treated males. Hindrance of SNBP function is expected to cause defective paternal nuclear behavior, a prediction that would be consistent with the altered sex ratio when this gene is targeted. Specifically, at 0 to 1 h after fertilization, in a number of embryos, the sperm- and egg-derived nuclei have moved next to each other in the egg's cytoplasm, having entered the first mitotic division. In embryos produced by untreated males (not shown) or Nv-PL2-treated males, the 2 nuclei looked similar in their level of chromatin condensation, with the exception that sometimes the paternal nuclear material looked less granular, likely due to the unique chromatin repackaging that it undergoes during the SNBP-to-histone transition (*n* = 7/8 embryos from sRNAi-Nv-PL1 males) (Fig. 3c). This observation is consistent with subtle differences between the egg- and sperm-derived nuclei at this time in *D. melanogaster* (Loppin et al. 2015). In contrast, in approximately one-third of embryos produced by sRNAi-Nv-PL1 males, the paternal chromatin appeared irregularly shaped and undercondensed, indicating defects in the chromatin state (*n* = 7/20 embryos) (Fig. 3c). In the most extreme case, in 1 embryo, a completely uncondensed sperm nucleus was positioned immediately beside the egg-derived chromosomes during metaphase (Supplementary Fig. 3). These observations are strongly suggestive that Nv-PL1 functions as an SNBP. Finally, mature sperm from males sRNAi treated for each of these genes showed no visible morphological defects (Fig. 3d), suggesting that the perturbations to chromatin are subtle and do not visibly affect other aspects of spermatogenesis.

## Discussion

Here, we used a computational/bioinformatic approach to identify SNBPs in the jewel wasp *N. vitripennis*. This approach is independent of and complementary to the biochemical approach that was previously employed in this insect and others for the same purpose (Leyden et al. 2024). By comparing previously published transcriptomes and subsequent filtering with specific SNBP criteria, we ended up with a list of 13 genes that (i) are expressed exclusively in the male reproductive tract, (ii) are moderately to highly basic, and (iii) have GO terms that pertain to DNA and chromatin. The 2 highest-expressed wasp genes in this list match arginine- and lysine-rich peptides that were biochemically isolated from male wasp reproductive tracts (Leyden et al. 2024). For this reason, we considered these genes to be the strongest SNBP candidates, thus warranting further consideration (discussed below). Of the other 11 genes, 2 have SNBP-like characteristics, but they were not identified biochemically, perhaps due to lower protein abundance levels. The remaining 9 genes encode proteins that, upon closer inspection, were found to have predicted structures, sizes, and functional motifs or corresponding mRNA expression levels that deviate from those expected of SNBPs, arguing that they may have other roles in chromatin or other sperm-specific processes.

The 2 top candidate SNBP genes in our study, named Nv-PL1 and Nv-PL2, encode proteins with PL properties. Each protein contains a WH domain near the N-terminus, while the remainder is unstructured. Within the unstructured regions reside the highly basic peptides that were previously identified biochemically from the reproductive tracts of adult males. Thus, like other PLs (Carlos et al. 1993; Lewis et al. 2004b; D’Ippolito et al. 2019), these *Nasonia* proteins are likely proteolytically cleaved to produce functional peptides that may help achieve the final, hypercondensed state of sperm chromatin. The results of our sRNAi experiments further support the identity of Nv-PL1 as a PL. Specifically, degradation of the transcripts from this gene caused a range of subtle to severe defects in sperm nuclear morphology during the first embryonic mitotic division and a change in brood sex ratio, which is normally highly female biased, toward males. Despite the presence of sperm chromatin defects in some embryos, a number of other embryos sired by sRNAi-treated males appeared relatively normal in nuclear morphology. This modest effect on chromatin morphology among all examined embryos may stem from the fact that sRNAi targeting of Nv-PL1 resulted in only a 50% transcript reduction efficiency, thus conveying a hypomorphic effect. Additionally, the sRNAi treatment is likely variable across treated males and individual sperm. Finally, Nv-PL1 is one of the highest-expressed genes in the testis, likely limiting the maximum level of transcript degradation that can be reasonably achieved. Although we were able to obtain an ∼80% degradation efficiency for Nv-PL2 transcripts, we saw only a small shift in the sex ratio of F1 progeny and no strong effect on sperm chromatin morphology. Considering these results, we speculate that Nv-PL1 may be the more important SNBP of these 2 PLs. This possibility is consistent with the fact that Nv-PL1 transcripts are ∼5-fold more abundant in the testis than those expressed by Nv-PL2.

Nv-PL1 is highly similar to PpH1V1, a previously characterized protein in another parasitoid wasp, *P. puparum*. Like Nv-PL1, treatment of the PpH1V1 gene with sRNAi resulted in a similar male-biasing effect of progeny broods. This similarity in sequence and phenotype strongly suggests that these 2 genes are orthologs. When PpH1V1 was described, it was referred to as a histone H1 variant (Yuan et al. 2023). Indeed, the corresponding gene was discovered by bioinformatically searching the *P. puparum* genome for homologs using histone H1 as a query sequence (Yuan et al. 2023). Given the orthologous relationship of PpH1V1 and Nv-PL1, the identity of Nv-PL1 as a PL, and the likelihood that PL genes are evolutionary derivatives of the histone H1 family (Lewis et al. 2004b; Eirín-López et al. 2006b), we offer that PpH1V1 may also be a PL. Future testing of this prediction could involve biochemically searching for PL peptides processed from PpH1V1 in mature sperm chromatin. Using immuno-fluorescence microscopy, it was shown that PpH1V1 was visible in elongating spermatid nuclei but not in the nuclei of mature sperm (Yuan et al. 2023). Such a result may reflect the removal of the N-terminal half of PpH1V1 if it is processed like Nv-PL1 and the PLs of other organisms. Alternatively, this observation may result from inaccessibility of PpH1V1 to immuno-fluorescence reagents in the highly condensed chromatin of mature sperm.

The small set of *Nasonia* SNBP genes stands in contrast to the greater number of TS HMG-box genes present in the genomes of *D. melanogaster* and other *Drosophila* species (Chang et al. 2023). We identified only 1 HMG-box gene that is expressed uniquely in the wasp testis. This gene is not considered to be a top SNBP candidate because it was not previously identified biochemically. However, we cannot rule out the possibility that the encoded protein may be a low-abundance SNBP or, alternatively, a transition protein that helps the exchange of histones for SNBPs. In *D. melanogaster*, Mst35A and Mst35B are broadly considered to be the main “Prots” of this organism. Indeed, they are synonymously referred to as Prots ProtA and ProtB, respectively, despite them having no structural similarity to true Prots (Jayaramaiah Raja and Renkawitz-Pohl 2005; Rathke et al. 2010; Kanippayoor and Moehring 2012; Gingell and McLean 2020). The idea that these genes encode SNBPs in the fruit fly is supported by elegant genetic analyses showing the presence of their encoded proteins in mature sperm chromatin (Jayaramaiah Raja and Renkawitz-Pohl 2005). Interestingly, however, males homozygous for loss-of-function mutations in either or both genes together are fertile, although their sperm do show some abnormal morphology and reduction in number (Rathke et al. 2010; Tirmarche et al. 2014). This lack of a strong fertility effect may reflect the possibility that these genes are functionally redundant with other SNBPs. Alternatively, they may play minor roles in sperm chromatin packaging. We note that *D. melanogaster* contains another male-specific gene, Mst77F, which was first characterized as a histone H1 family member, presumably because its encoded protein has a globular head region containing a WH-like domain and a largely unstructured C-terminal half (Russell and Kaiser 1993; Kost et al. 2015). Unlike Mst35A and Mst35B, males that are mutant for Mst77F are strongly sterile (Jayaramaiah Raja and Renkawitz-Pohl 2005; Kimura and Loppin 2016), which is to be expected for an important SNBP that is not functionally redundant with other SNBPs. Moreover, the Mst77F protein was found to be posttranslationally cleaved during spermatogenesis, with some part of the C-terminal half being incorporated into sperm chromatin (Kimura and Loppin 2016), similar to PLs. We propose that Mst77F may be the primary PL-type SNBP, or one of a few main ones, in *D. melanogaster*, with the HMG-box genes such as Mst35A and Mst35B being secondary (less functionally important) SNBPs. Moreover, while the array of SNBPs in *D. melanogaster* may be more numerous than in *N. vitripennis*, these PLs (Nv-PL1 and Nv-PL2 in *N. vitripennis* and Mst77F in *D. melanogaster*) may play a more central role in sperm chromatin condensation in both of these distantly related insects.

In contrast to the male-specific HMG-box genes in *Drosophila* species (Chang et al. 2023), Nv-PL1 and Nv-PL2 do not appear to be evolutionarily dynamic in copy number; each gene is present in single copy in *N. vitripennis* and other wasp species. This finding is consistent with the idea that conflict between the sex chromosomes may be a driving force in the rapid expansion and loss of HMG-box genes, especially those that are X and Y linked, in insects such as *Drosophila* species that possess sex chromosomes (Chang et al. 2023). Mst77F is also rapidly evolving, an effect that helps to suppress the uneven appearance of Y-bearing sperm (Chang et al. 2025). Indeed, *N. vitripennis* and other hymenopteran insects do not have sex chromosomes. Instead, the 2 sexes in hymenopterans differ genotypically only by the number of chromosome sets (1 in males vs 2 in females). We note that, because our study did not involve extensive analysis of SNBP genes in other hymenopteran insects, we cannot rule out the possibility of more complicated SNBP dynamics in this group.

While the 2 wasp PLs are not evolutionarily dynamic in copy number, their C-terminal halves have substantial amino acid differences, including in the PL peptide region, among homologs. In this regard, the PL peptides may be similar to Prots in other organisms that exhibit rapid evolution in their protein sequences (Torgerson et al. 2002; Lüke et al. 2014; Chang et al. 2023). From the work presented here, we cannot say whether the PL peptides of the jewel wasp are under positive selection, though preliminary evidence suggests that the WH domain of Nv-PL2 in particular may be evolving adaptively. Given the limited number of detectable orthologs and the inability to reliably align the peptide region, future analyses may benefit from employing population-level approaches to test for positive selection. However, the visible changes in PL peptide sequences may be driven by selective pressures on sperm nuclear shape that have been observed for Prots in vertebrates (Lüke et al. 2014). Alternatively, changes in PL peptide sequences may result from pressures imposed by certain strains of *Wolbachia*, intracellular bacteria that induce a chromatin-altering activity known as cytoplasmic incompatibility (CI) (Hochstrasser 2023). CI-causing *Wolbachia* bacteria express 2 cytoplasmic incompatibility factors (Cif proteins), one which becomes integrated into sperm chromatin and plays a role in its destruction (Hochstrasser 2023). This disruption is a facet of CI that ultimately gives a transmission advantage to the *Wolbachia*, but it may impose longer-term costs to the insect host. Work from a previous study suggested that CI-causing *Wolbachia* may disrupt aspects of the protamine-to-histone transition following fertilization (Landmann et al. 2009). It is possible that certain amino acid changes in PLs and other proteins that function in this process may be advantageous to the insect, leading to suppression of CI strength and, thus, increased insect fitness and fecundity. Our observations support these potential explanations and future studies aimed at elucidating the underlying details.

## Supplementary Material

jkag066_Supplementary_Data

## Data Availability

All unpublished data associated with this manuscript are included within the accompanying files. Published transcriptome data can be found in the following references: Ferree et al. 2015 and Dalla Benetta et al. 2020. Supplemental material available at *G3* online.

## References

[jkag066-B1] Akbari OS, Antoshechkin I, Hay BA, Ferree PM. 2013. Transcriptome profiling of *Nasonia vitripennis* testis reveals novel transcripts expressed from the selfish B chromosome, paternal sex ratio. G3 (Bethesda). 3:1597–1605. 10.1534/g3.113.007583. 23893741 PMC3755920

[jkag066-B2] Alder D, Gorovsky MA. 1975. Electrophoretic analysis of liver and testis histones of the frog Rana pipiens. J Cell Biol. 64:389–397. 10.1083/jcb.64.2.389. 1078823 PMC2109491

[jkag066-B3] Álvarez-Carretero S, Kapli P, Yang Z. 2023. Beginner's guide on the use of PAML to detect positive selection. Mol Biol Evol. 40:msad041. 10.1093/molbev/msad041. 37096789 PMC10127084

[jkag066-B4] Alvi ZA, Chu T-C, Schawaroch V, Klaus AV. 2013. Protamine-like proteins in 12 sequenced species of *Drosophila*. Protein Pept Lett. 20:17–35. 10.2174/092986613804096847. 22789106

[jkag066-B5] Ausio J et al 1987. Structural characterization of the trypsin-resistant core in the nuclear sperm-specific protein from Spisula solidissima. Biochemistry. 26:975–982. 10.1021/bi00378a001. 3567164

[jkag066-B6] Ausió J . 1999. Histone H1 and evolution of sperm nuclear basic proteins. J Biol Chem. 274:31115–31118. 10.1074/jbc.274.44.31115. 10531297

[jkag066-B7] Balhorn R . 1982. A model for the structure of chromatin in mammalian sperm. J Cell Biol. 93:298–305. 10.1083/jcb.93.2.298. 7096440 PMC2112839

[jkag066-B8] Balhorn R . 2007. The protamine family of sperm nuclear proteins. Genome Biol. 8:227. 10.1186/gb-2007-8-9-227. 17903313 PMC2375014

[jkag066-B9] Bloch DP . 1976. Histones of sperm. In: King RC, editor. Handbook of genetics. New York: Plenum Press. p. 139–167.

[jkag066-B10] Braun RE . 2001. Packaging paternal chromosomes with protamine. Nat Genet. 28:10–12. 10.1038/ng0501-10. 11326265

[jkag066-B11] Buchfink B, Reuter K, Drost H-G. 2021. Sensitive protein alignments at tree-of-life scale using DIAMOND. Nat Methods. 18:366–368. 10.1038/s41592-021-01101-x. 33828273 PMC8026399

[jkag066-B12] Carlos S, Hunt DF, Rocchini C, Arnott DP, Ausio J. 1993. Post-translational cleavage of a histone H1-like protein in the sperm of Mytilus. J Biol Chem. 268:195–199. 10.1016/S0021-9258(18)54133-8. 8416927

[jkag066-B13] Chang C-H, de la Cruz AF, Natividad IM, Noyola A, Malik HS. 2025. Rapid protamine evolution suppresses meiotic drive in *Drosophila* [preprint]. bioRxiv. 10.1101/2025.09.03.674087.

[jkag066-B14] Chang C-H, Mejia Natividad I, Malik HS. 2023. Expansion and loss of sperm nuclear basic protein genes in correspond with genetic conflicts between sex chromosomes. Elife. 12:e85249. 10.7554/eLife.85249. 36763410 PMC9917458

[jkag066-B15] Dalla Benetta E et al 2020. Genome elimination mediated by gene expression from a selfish chromosome. Sci Adv. 6:eaaz9808. 10.1126/sciadv.aaz9808. 32284986 PMC7124933

[jkag066-B16] D’Ippolito RA et al 2019. Protamines from liverwort are produced by post-translational cleavage and C-terminal di-aminopropanelation of several male germ-specific H1 histones. J Biol Chem. 294:16364–16373. 10.1074/jbc.RA119.010316. 31527083 PMC6827293

[jkag066-B17] Dubruille R et al 2023. Histone removal in sperm protects paternal chromosomes from premature division at fertilization. Science. 382:725–731. 10.1126/science.adh0037. 37943933 PMC11180706

[jkag066-B18] Dubruille R, Horard B, Loppin B. 2025. A haystack in the needle: packaging sperm DNA in insects. Curr Opin Genet Dev. 93:102378. 10.1016/j.gde.2025.102378. 40627886

[jkag066-B19] Eirín-López JM, Frehlick LJ, Ausió J. 2006a. Protamines, in the footsteps of linker histone evolution. J Biol Chem. 281:1–4. 10.1074/jbc.R500018200. 16243843

[jkag066-B20] Eirín-López JM, Lewis JD, Howe LA, Ausió J. 2006b. Common phylogenetic origin of protamine-like (PL) proteins and histone H1: evidence from bivalve PL genes. Mol Biol Evol. 23:1304–1317. 10.1093/molbev/msk021. 16613862

[jkag066-B21] Ferree PM et al 2015. Identification of genes uniquely expressed in the germ-line tissues of the jewel wasp *Nasonia vitripennis*. G3 (Bethesda). 5:2647–2653. 10.1534/g3.115.021386. 26464360 PMC4683638

[jkag066-B22] Ferree PM, Cummings J, Garman E, Solomon J, Martinez KS. 2026. A male-transmitted B chromosome undergoes strong meiotic drag in females of the jewel wasp *Nasonia vitripennis*. PLoS Biol. 24:e3003599. 10.1371/journal.pbio.3003599. 41544124 PMC12826520

[jkag066-B23] Fortes MRS et al 2014. Sperm protamine deficiency correlates with sperm DNA damage in *Bos indicus* bulls. Andrology. 2:370–378. 10.1111/j.2047-2927.2014.00196.x. 24634207

[jkag066-B24] Gingell LF, McLean JR. 2020. A protamine knockdown mimics the function of in *Sd* in *Drosophila melanogaster*. G3 (Bethesda). 10:2111–2115. 10.1534/g3.120.401307. 32321837 PMC7263674

[jkag066-B25] GitHub—lh3/seqtk: Toolkit for processing sequences in FASTA/Q formats GitHub . 2025. https://github.com/lh3/seqtk.

[jkag066-B26] Gotz S et al 2008. High-throughput functional annotation and data mining with the Blast2GO suite. Nucleic Acids Research. 36:3420–3435. 10.1093/nar/gkn176. 18445632 PMC2425479

[jkag066-B27] Hake SB, Allis CD. 2006. Histone H3 variants and their potential role in indexing mammalian genomes: the “H3 barcode hypothesis”. Proc Natl Acad Sci U S A. 103:6428–6435. 10.1073/pnas.0600803103. 16571659 PMC1564199

[jkag066-B28] Hartman PG, Chapman GE, Moss T, Bradbury EM. 1977. Studies on the role and mode of operation of the very-lysine-rich histone H1 in eukaryote chromatin. The three structural regions of the histone H1 molecule. Eur J Biochem. 77:45–51. 10.1111/j.1432-1033.1977.tb11639.x. 908338

[jkag066-B29] Hochstrasser M . 2023. Molecular biology of cytoplasmic incompatibility caused by *Wolbachia* endosymbionts. Annu Rev Microbiol. 77:299–316. 10.1146/annurev-micro-041020-024616. 37285552

[jkag066-B30] Jacob Y et al 2014. Selective methylation of histone H3 variant H3.1 regulates heterochromatin replication. Science. 343:1249–1253. 10.1126/science.1248357. 24626927 PMC4049228

[jkag066-B31] Jayaramaiah Raja S, Renkawitz-Pohl R. 2005. Replacement by *Drosophila melanogaster* protamines and Mst77F of histones during chromatin condensation in late spermatids and role of sesame in the removal of these proteins from the male pronucleus. Mol Cell Biol. 25:6165–6177. 10.1128/MCB.25.14.6165-6177.2005. 15988027 PMC1168805

[jkag066-B32] Jones P et al 2014. InterProScan 5: genome-scale protein function classification. Bioinformatics. 30:1236–1240. 10.1093/bioinformatics/btu031. 24451626 PMC3998142

[jkag066-B33] Jumper J et al 2021. Highly accurate protein structure prediction with AlphaFold. Nature. 596:583–589. 10.1038/s41586-021-03819-2. 34265844 PMC8371605

[jkag066-B34] Kanippayoor RL, Moehring AJ. 2012. Allelic expression of *Drosophila* protamines during spermatogenesis. Int J Evol Biol. 2012:947381. 10.1155/2012/947381. 22567534 PMC3332191

[jkag066-B35] Kasinsky HE, Gowen BE, Ausió J. 2021. Spermiogenic chromatin condensation patterning in several hexapods may involve phase separation dynamics by spinodal decomposition or microemulsion inversion (nucleation). Tissue Cell. 73:101648. 10.1016/j.tice.2021.101648. 34537592

[jkag066-B36] Katoh K, Standley DM. 2013. MAFFT multiple sequence alignment software version 7: improvements in performance and usability. Mol Biol Evol. 30:772–780. 10.1093/molbev/mst010. 23329690 PMC3603318

[jkag066-B37] Kimura S, Loppin B. 2016. The *Drosophila* chromosomal protein Mst77F is processed to generate an essential component of mature sperm chromatin. Open Biol. 6:160207. 10.1098/rsob.160207. 27810970 PMC5133442

[jkag066-B38] Kost N et al 2015. Multimerization of *Drosophila* sperm protein Mst77F causes a unique condensed chromatin structure. Nucleic Acids Res. 43:3033–3045. 10.1093/nar/gkv015. 25735749 PMC4381051

[jkag066-B39] Kozlowski LP . 2016. IPC—isoelectric point calculator. Biol Direct. 11:55. 10.1186/s13062-016-0159-9. 27769290 PMC5075173

[jkag066-B40] Landmann F, Orsi GA, Loppin B, Sullivan W. 2009. Wolbachia-mediated cytoplasmic incompatibility is associated with impaired histone deposition in the male pronucleus. PLoS Pathog. 5:e1000343. 10.1371/journal.ppat.1000343. 19300496 PMC2652114

[jkag066-B41] Lewis JDM . 2003. Histone H1 and the evolution of protamines. 10.1073/pnas.0308721101.

[jkag066-B42] Lewis JD et al 2004a. All roads lead to arginine: the squid protamine gene. J Mol Evol. 58:673–680. 10.1007/s00239-004-2589-8. 15461424

[jkag066-B43] Lewis JD et al 2004b. Histone H1 and the origin of protamines. Proc Natl Acad Sci U S A. 101:4148–4152. 10.1073/pnas.0308721101. 15024099 PMC384709

[jkag066-B44] Leyden MR et al 2024. Protamines and the sperm nuclear basic proteins Pandora's box of insects. Biochem Cell Biol. 102:238–251. 10.1139/bcb-2023-0363. 38408323 PMC12216123

[jkag066-B45] Li M et al 2017. Generation of heritable germline mutations in the jewel wasp *Nasonia vitripennis* using CRISPR/Cas9. Sci Rep. 7:901. 10.1038/s41598-017-00990-3. 28424460 PMC5430486

[jkag066-B46] Loppin B, Dubruille R, Horard B. 2015. The intimate genetics of Drosophila fertilization. Open Biol. 5:150076. 10.1098/rsob.150076. 26246493 PMC4554920

[jkag066-B47] Lüke L, Tourmente M, Dopazo H, Serra F, Roldan ERS. 2016a. Selective constraints on protamine 2 in primates and rodents. BMC Evol Biol. 16:21. 10.1186/s12862-016-0588-1. 26801756 PMC4724148

[jkag066-B48] Lüke L, Tourmente M, Roldan ERS. 2016b. Sexual selection of protamine 1 in mammals. Mol Biol Evol. 33:174–184. 10.1093/molbev/msv209. 26429923

[jkag066-B49] Lüke L, Vicens A, Tourmente M, Roldan ERS. 2014. Evolution of protamine genes and changes in sperm head phenotype in rodents. Biol Reprod. 90:67. 10.1095/biolreprod.113.115956. 24522148

[jkag066-B50] Lynch JA, Desplan C. 2006. A method for parental RNA interference in the wasp *Nasonia vitripennis*. Nat Protoc. 1:486–494. 10.1038/nprot.2006.70. 17406271

[jkag066-B51] Malarkey CS, Churchill MEA. 2012. The high mobility group box: the ultimate utility player of a cell. Trends Biochem Sci. 37:553–562. 10.1016/j.tibs.2012.09.003. 23153957 PMC4437563

[jkag066-B52] Nili HA, Mozdarani H, Aleyasin A. 2009. Correlation of sperm DNA damage with protamine deficiency in Iranian subfertile men. Reprod Biomed Online. 18:479–485. 10.1016/S1472-6483(10)60123-X. 19400988

[jkag066-B53] Rathke C et al 2010. Distinct functions of Mst77F and protamines in nuclear shaping and chromatin condensation during Drosophila spermiogenesis. Eur J Cell Biol. 89:326–338. 10.1016/j.ejcb.2009.09.001. 20138392

[jkag066-B54] Rathke C, Baarends WM, Awe S, Renkawitz-Pohl R. 2014. Chromatin dynamics during spermiogenesis. Biochim Biophys Acta. 1839:155–168. 10.1016/j.bbagrm.2013.08.004. 24091090

[jkag066-B55] Rice P, Longden I, Bleasby A. 2000. EMBOSS: the European Molecular Biology Open Software Suite. Trends Genet. 16:276–277. 10.1016/S0168-9525(00)02024-2. 10827456

[jkag066-B56] Rousseaux S et al 2008. Epigenetic reprogramming of the male genome during gametogenesis and in the zygote. Reprod Biomed Online. 16:492–503. 10.1016/S1472-6483(10)60456-7. 18413057

[jkag066-B57] Russell SR, Kaiser K. 1993. Drosophila melanogaster male germ line-specific transcripts with autosomal and Y-linked genes. Genetics. 134:293–308. 10.1093/genetics/134.1.293. 8514138 PMC1205432

[jkag066-B58] Saperas N et al 2006. A unique vertebrate histone H1-related protamine-like protein results in an unusual sperm chromatin organization. FEBS J. 273:4548–4561. 10.1111/j.1742-4658.2006.05461.x. 16965539

[jkag066-B59] Saperas N, Ausio J, Lloris D, Chiva M. 1994. On the evolution of protamines in bony fish: alternatives to the “retroviral horizontal transmission” hypothesis. J Mol Evol. 39:282–295. 10.1007/BF00160152. 7932790

[jkag066-B60] Suyama M, Torrents D, Bork P. 2006. PAL2NAL: robust conversion of protein sequence alignments into the corresponding codon alignments. Nucleic Acids Res. 34:W609–W612. 10.1093/nar/gkl315. 16845082 PMC1538804

[jkag066-B61] Tirmarche S et al 2014. Drosophila protamine-like Mst35Ba and Mst35Bb are required for proper sperm nuclear morphology but are dispensable for male fertility. G3 (Bethesda). 4:2241–2245. 10.1534/g3.114.012724. 25236732 PMC4232549

[jkag066-B62] Torgerson DG, Kulathinal RJ, Singh RS. 2002. Mammalian sperm proteins are rapidly evolving: evidence of positive selection in functionally diverse genes. Mol Biol Evol. 19:1973–1980. 10.1093/oxfordjournals.molbev.a004021. 12411606

[jkag066-B63] Werren JH, Loehlin DW. 2009. The parasitoid wasp Nasonia: an emerging model system with haploid male genetics. Cold Spring Harb Protoc. 2009:db.emo134. 10.1101/pdb.emo134. PMC2916733 20147035

[jkag066-B64] Yang Z . 1997. PAML: a program package for phylogenetic analysis by maximum likelihood. Comput Appl Biosci. 13:555–556. 10.1093/bioinformatics/13.5.555. 9367129

[jkag066-B65] Yuan B et al 2023. A rapidly evolving single copy histone H1 variant is associated with male fertility in a parasitoid wasp. Front Cell Dev Biol. 11:1166517. 10.3389/fcell.2023.1166517. 37325562 PMC10264595

[jkag066-B66] Zhang X, Singh A, Soriano Martinez K, Ferree PM. 2024. Direct parental (DIPA) CRISPR in the jewel wasp, *Nasonia vitripennis*. G3 (Bethesda). 14:jkae095. 10.1093/g3journal/jkae095. 38734969 PMC11228858

