# Supplementary material for: The jewel wasp Nasonia vitripennis utilizes two single-copy, protamine-like sperm nuclear basic proteins
Source: G3 (Bethesda). 2026 Mar 20;16(5):jkag066. doi: 10.1093/g3journal/jkag066 (PMC13148410; doi:10.1093/g3journal/jkag066)
Supplement: jkag066_Supplementary_Data [file jkag066_supplementary_data.zip › Supplemental_Material_Legends_G3-2026-406662.docx]

**Supplemental Figure Legends and Table Titles**

**Supplemental Table 1**. Filtering steps leading to top SNBP candidates

**Supplemental Figure 1**. Two *N. vitripennis* SNBP secondary candidates. (A) LOC103316131 has a PL-like structure, including a WH-like domain (black box) and an arginine/lysine-rich region analogous to the PL-peptide region in Nv-PL1 and Nv-PL2 (red box). LOC 103316943 contains an HMG-box domain (green). Arginine and lysine residues are shown in red font. (B) The AlphaFold-predicted 3D structures are shown for each of these proteins.

**Supplemental Figure 2**. Alignments of the WH domain and the PL-peptide for both Nv-PL1 and Nv-PL2 across selected hymenopteran orthologs. The layout here is the same as for Figure 2. The full genus and species names for the Hymenopteran PL orthologs are *Apis mellifera (Amel), Bombus impatiens (Bmip), Bombus terrestris (Bter), Cephus cinctus (Ccin), Linepithema humile (Lhum), Megachile rotundata (Mrot), Mischocttarus mexicanus (Mmex), Monomorium pharaonis (Mpha), Nasonia vitripennis (Nvit), Nomia melanderia (Nmel), Pogonomyrmex barbatus (Pbar), Trichogramma brassicae (Tbra), Trichogramma pretiosum, Trichomalopsis sarcophage (Tsar), (Tpre), Vespa mandarinia (Vman), Vespula pensylvanica (Vpen), Vespa velutina (Vvel), Wasmannia auropunctata (Waur).*

**Supplemental Figure 3**. An image taken in a young embryo produced by an sRNAi-Nv-PL1-treated father. In this embryo, the sperm chromatin (left) has remained completely condensed, retaining its sperm nuclear appearance. The egg’s nuclear material (right) has entered mitosis, evident by the individualized chromosomes. Normally both paternal and maternal sets transform together into individualized chromosomes as they enter the first division.

**Supplemental Method**. Python scripts used to determine protein sequence length, amino acid composition, and arginine placement.
