# Supplementary material for: The jewel wasp Nasonia vitripennis utilizes two single-copy, protamine-like sperm nuclear basic proteins
Source: G3 (Bethesda). 2026 Mar 20;16(5):jkag066. doi: 10.1093/g3journal/jkag066 (PMC13148410; doi:10.1093/g3journal/jkag066)
Supplement: jkag066_Supplementary_Data [file jkag066_supplementary_data.zip › Supplemental_Methods_G3-2026-406662.docx]

Supplementary Method: Python scripts used to determine protein sequence length, amino acid composition, and arginine placement

import pandas as pd

import datetime

print(str(datetime.datetime.now()) + ": started")

startTime = datetime.datetime.now()

f = open(r"protein.faa", "r")

df = pd.DataFrame()

names = []

listPercentR = []

listPercentK = []

#Identifying protein sequence length, amino acid composition, and arginine/lysine placement

def countR(seq):

rCount = seq.count("R")

totalLength = len(seq)

calcPercentR = (rCount/totalLength) * 100

return calcPercentR

def countK(seq):

kCount = seq.count("K")

totalLength = len(seq)

calcPercentK = (kCount/totalLength) * 100

return calcPercentK

i = 0

aaSeq = ""

for line in f:

if line[0] == ">" and str(line) != ">end":

#counter

i += 1

if i % 500 == 0:

print(i)

#add name

line = line.replace("\n", '')

line = line.replace(">", '')

geneNP = line.split(sep=" ")[0]

names.append(geneNP)

#add percents

if aaSeq != "": #exculde first case

listPercentR.append(countR(aaSeq))

listPercentK.append(countK(aaSeq))

aaSeq = ""

elif str(line) == ">end":

listPercentR.append(countR(aaSeq))

listPercentK.append(countK(aaSeq))

else:

line = line.replace("\n", '')

aaSeq += line

df["Gene NP"] = names

df["% Arginine"] = listPercentR

df["% Lysine"] = listPercentK

print(df)

df.to_csv(r"percents.csv", index=False)

endTime = datetime.datetime.now()

print(str(endTime) + ": finished")

timeDiff = endTime - startTime

print("Took " + str(timeDiff))
