# Supplementary figures and images for: The jewel wasp Nasonia vitripennis utilizes two single-copy, protamine-like sperm nuclear basic proteins
Source: G3 (Bethesda). 2026 Mar 20;16(5):jkag066. doi: 10.1093/g3journal/jkag066 (PMC13148410; doi:10.1093/g3journal/jkag066)

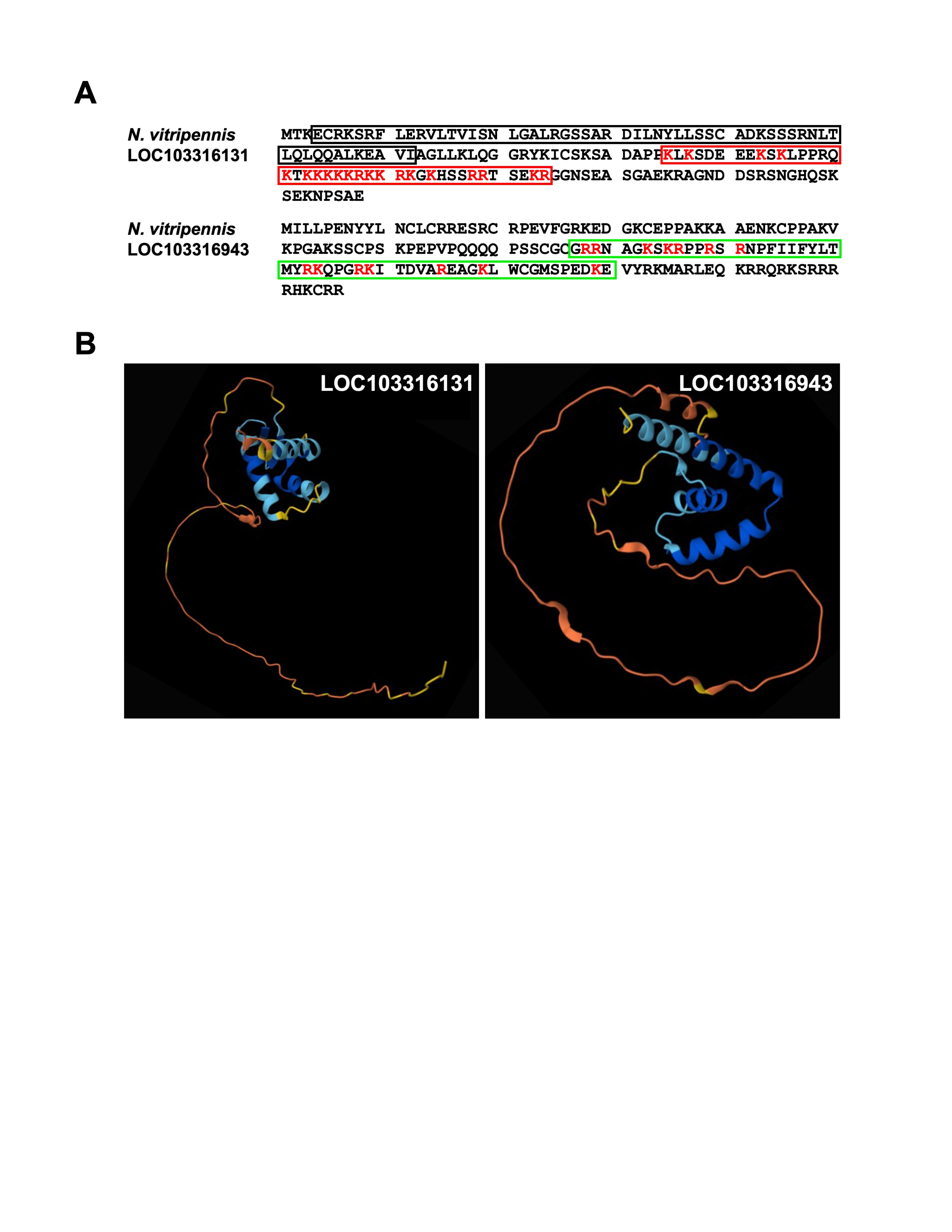

Supplement: jkag066_Supplementary_Data [file jkag066_supplementary_data.zip › Supplemental_Figure_1_G3-2026-406662.png]

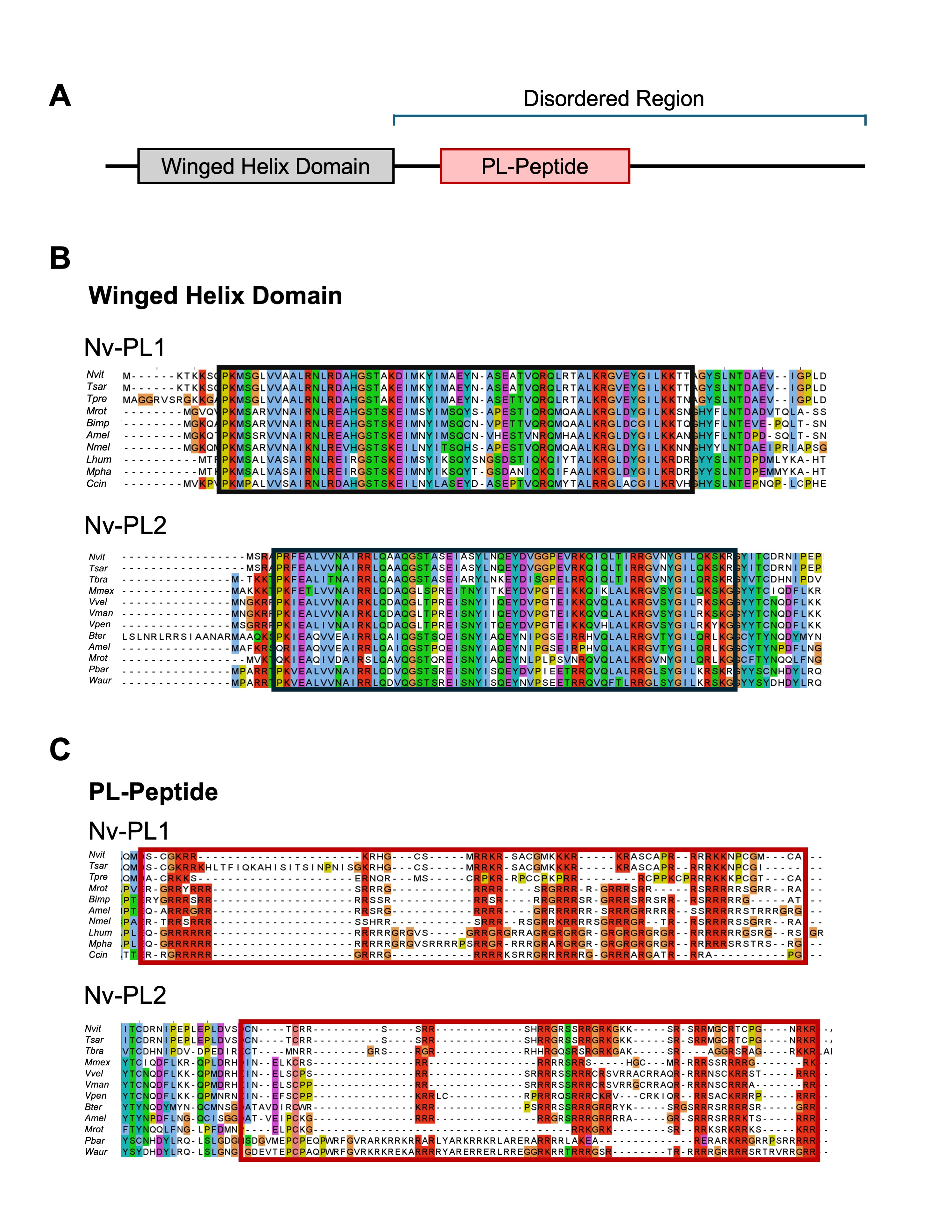

Supplement: jkag066_Supplementary_Data [file jkag066_supplementary_data.zip › Supplemental_Figure_2_G3-2026-406662.png]

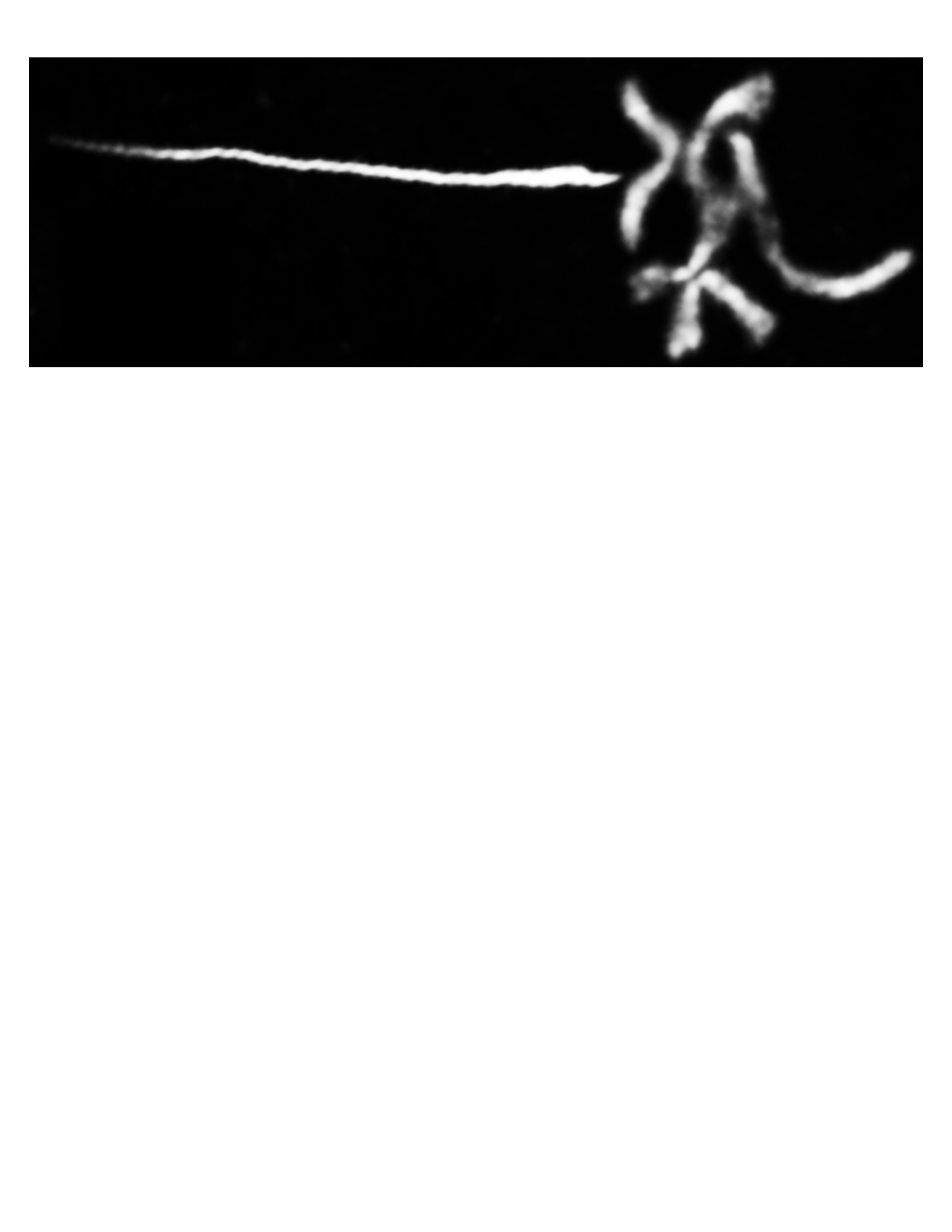

Supplement: jkag066_Supplementary_Data [file jkag066_supplementary_data.zip › Supplemental_Figure_3_G3-2026-406662.png]
